# Supplementary material for: A UMLS-based spell checker for natural language processing in vaccine safety
Source: BMC Med Inform Decis Mak. 2007 Feb 12;7:3. doi: 10.1186/1472-6947-7-3 (PMC1805499; doi:10.1186/1472-6947-7-3)
Supplement: Additional file 13 — RAP application directory. Sets up directory for RAP (RDF application for PHP) source code [file 1472-6947-7-3-S13.gz › rap/doc/tests/test_SetDatabaseConnection.php.txt]

Test Store Models in Database

php
// Include RAP
define("RDFAPI\_INCLUDE\_DIR", "./../api/");
include(RDFAPI\_INCLUDE\_DIR . "RDFAPI.php");
## 1. Connect to MsAccess database (via ODBC) and create tables.
// Connect to MsAccess (rdf\_db DSN) database using connection settings
// defined in constants.php :
// ----------------------------------------------------------------------------------
// Database
// ----------------------------------------------------------------------------------
// define("ADODB\_DB\_DRIVER", "odbc");
// define("ADODB\_DB\_HOST", "rdf\_db");
// define("ADODB\_DB\_NAME", "");
// define("ADODB\_DB\_USER", "");
// define("ADODB\_DB\_PASSWORD", "");
$rdf\_database = ModelFactory::getDbStore();
// Create tables for MsAccess
$rdf\_database-createTables('MsAccess');
## 2. Connect to MySQL database and create tables.
/\*
// Connect to MySQL database with user defined connection settings
$rdf\_database = ModelFactory::getDbStore('MySQL', 'localhost', 'db\_name', 'user\_name', 'password' );
// Create tables for MySQL
$rdf\_database->createTables('MySQL');
\*/
## 3. Connect to other databases
/\*
// Example:
// Connect to Oracle database with user defined connection settings
$rdf\_database = ModelFactory::getDbStore('Oracle', FALSE, 'db\_name', 'username', 'password' );
// Note: Method createTables() currently supports only MySQL and MsAccess.
// If you want to use other databases, you will have to create tables by yourself
// according to the abstract database schema described in the API documentation.
\*/
## 4. Close the database connection
// Close the connection
$rdf\_database->close();
?>
